# Supplementary material for: Characterization of a heme-degrading enzyme that mediates fitness and pathogenicity in Enterococcus faecalis
Source: mBio. 2025 Apr 11;16(5):e00146-25. doi: 10.1128/mbio.00146-25 (PMC12077173; doi:10.1128/mbio.00146-25)
Supplement: Supplemental material — Supplemental figures and tables. [file mbio.00146-25-s0001.docx]

**Table S1.** Table shows proteins used for sequence alignment against RS05575 (WP_025188678.1) and their respective percent identity and similarities.

| **Species** | **Protein** | **Identity (%)** | **Similarity (%)** |
| --- | --- | --- | --- |
| *Fusobacterium nucleatum* ATCC 25586 | [WP_011016639.1](https://www.ncbi.nlm.nih.gov/sites/entrez?cmd=Search&db=protein&term=WP_011016639.1&dopt=GenBank) | 22.85 | 41.18 |
| *Escherichia coli* O157:H7 2159 | [WP_000993317.1](http://www.ncbi.nlm.nih.gov/protein/WP_000993317.1) | 23.47 | 44.53 |
| *Vibrio cholerae* 12129 | [EEO00035.1](https://www.ncbi.nlm.nih.gov/protein/EEO00035.1) | 24.08 | 36.66 |
| *Pseudomonas aeruginosa* M18 | [WP_014602385.1](https://www.ncbi.nlm.nih.gov/protein/WP_014602385.1) | 30.65 | 48.49 |
| *Staphylococcus saprophyticus* ATCC 15305 | WP_011302991.1 | 46.6 | 69.63 |
| *Staphylococcus aureus* NCTC 8325 | WP_000218088.1 | 46.86 | 67.8 |
| *Staphylococcus epidermidis* ATCC 12228 | WP_002468788.1 | 47.38 | 67.28 |
| *Staphylococcus lugdunenesis* M23590 | WP_002458976.1 | 48.86 | 67.54 |
| *Staphylococcus gallinarum* STP534 | [WP_107512989.1](https://www.ncbi.nlm.nih.gov/sites/entrez?cmd=Search&db=protein&term=WP_107512989.1&dopt=GenBank) | 48.95 | 69.9 |
| *Streptococcus pyogenes* MGAS5005 | WP_010922247.1 | 51.82 | 71.35 |
| *Streptococcus salivarius* DE0578 | WP_145516193.1 | 52.08 | 75.00 |
| *Streptococcus agalactiae* ATCC13813 | EFV97271.1 | 52.86 | 71.09 |
| *Streptococcus oralis* ATCC 35037 | [WP_001170059.1](http://www.ncbi.nlm.nih.gov/protein/WP_001170059.1) | 52.86 | 72.4 |
| *Streptococcus sanguinis* ATCC 29667 | WP_002924887.1 | 52.86 | 72.14 |
| *Streptococcus mutans* UA159 | WP_002352330.1 | 53.13 | 72.92 |
| *Streptococcus pneumoniae* TIGR4 | WP_001170072.1 | 53.13 | 72.92 |
| *Streptococcus mitis* ATCC 6249 | [WP_001170085.1](https://www.ncbi.nlm.nih.gov/sites/entrez?cmd=Search&db=protein&term=WP_001170085.1&dopt=GenBank) | 53.9 | 72.9 |
| *Enterococcus cecorum* ATCC 43198 | [WP_016252340.1](https://www.ncbi.nlm.nih.gov/sites/entrez?cmd=Search&db=protein&term=WP_016252340.1&dopt=GenBank) | 61.98 | 75.00 |
| *Melissococcus plutonius* ATCC 35311 | [WP_013774161.1](http://www.ncbi.nlm.nih.gov/protein/WP_013774161.1) | 65.00 | 81.00 |
| *Enterococcus gallinarum* CQFY22-081 | WP_317811148.1 | 66.5 | 78.68 |
| *Tetragenococcus halophilus* NBRC 114546 | [WP_284247512.1](https://www.ncbi.nlm.nih.gov/protein/WP_284247512.1) | 67.88 | 80.50 |
| *Enterococcus casseliflavus* ATCC 49996 | WP_010748104.1 | 68.43 | 79.29 |
| *Vagococcus fluvialis* bH819 | WP_086952342.1 | 70.32 | 83.96 |
| *Enterococcus durans 8L1-82* | WP_142422290.1 | 70.51 | 83.59 |
| *Enterococcus faecium* DO | [WP_002289534.1](https://www.ncbi.nlm.nih.gov/protein/WP_002289534.1) | 71.03 | 84.10 |
| *Enterococcus lactis* MG28 | WP_274875586.1 | 71.54 | 84.10 |
| *Enterococcus hirae* ATCC 9790 | [WP_010737743.1](https://www.ncbi.nlm.nih.gov/sites/entrez?cmd=Search&db=protein&term=WP_010737743.1&dopt=GenBank) | 71.54 | 85.13 |
| *Enterococcus ureilyticus DSM 102981* | [WP_069638559.1](https://www.ncbi.nlm.nih.gov/sites/entrez?cmd=Search&db=protein&term=WP_069638559.1&dopt=GenBank) | 76.42 | 87.56 |
| *Enterococcus faecalis* V583 | AAO81097.1 | 96.7 | 96.95 |

**Table S2.** FMC media formulation.

| **Component** | **Final Concentration** |
| --- | --- |
| KH_2_PO_4_ | 0.944 mg mL^-1^ |
| K_2_HPO_4_ | 0.61mg mL^-1^ |
| (NH_4_)_2_SO_4_ | 1.2 mg mL^-1^ |
| L-Aspartic Acid | 2.3 µg mL^-1^ |
| L-Phenylalanine | 0.2 mg mL^-1^ |
| L-Serine | 0.2 mg mL^-1^ |
| L-Proline | 0.4 mg mL^-1^ |
| L-Hydroxyproline | 0.4 mg mL^-1^ |
| Glycine | 8 µg mL^-1^ |
| Leucine | 8 µg mL^-1^ |
| L-Glutamic Acid | 6.5 µg mL^-1^ |
| DL-Alanine | 0.4 mg mL^-1^ |
| L-Isoleucine | 0.2 mg mL^-1^ |
| L-Methionine | 0.2 mg mL^-1^ |
| L-Threonine | 0.2 mg mL^-1^ |
| L-Arginine | 0.4 mg mL^-1^ |
| L-Histidine | 0.4 mg mL^-1^ |
| L-Tryptophan | 0.4 mg mL^-1^ |
| L-Valine | 0.2 mg mL^-1^ |
| L-Lysine | 0.552 mg mL^-1^ |
| Riboflavin | 8 µg mL^-1^ |
| P-aminobenzoic acid | 0.16 µg mL^-1^ |
| Thiamine HCl | 8 µg mL^-1^ |
| Nicotinamide | 4 µg mL^-1^ |
| Pantothenate | 1.72 µg mL^-1^ |
| Biotin | 0.02 µg mL^-1^ |
| Folic Acid | 0.2 µg mL^-1^ |
| Pyridoxal | 2.3 µg mL^-1^ |
| Glutamine | 10 µg mL^-1^ |
| L-Cystine | 0.4 mg mL^-1^ |
| L-Tyrosine | 0.4 mg mL^-1^ |
| Adenine | 0.06984 mg mL^-1^ |
| Guanine | 0.05448 mg mL^-1^ |
| Uracil | 0.06 mg mL^-1^ |
| MgSO_4_ 7H_2_O | 0.4 mg mL^-1^ |
| NaCl | 0.2 mg mL^-1^ |
| CaCl_2_ | 5.55 µg mL^-1^ |
| MnSO_4_ 7H_2_O | 10 µM |
| FeSO_4_ 7H_2_0 | 0 or 20 µM |
| NaOAcetate 3H_2_O | 6 mg mL^-1^ |
| Na_3_Citrate | 2.25 mg mL^-1^ |
| Na_2_CO_3_ H_2_O | 1.59 mg mL^-1^ |
| Glucose | 20 mM |

**Table S3.** Primers used in this study.

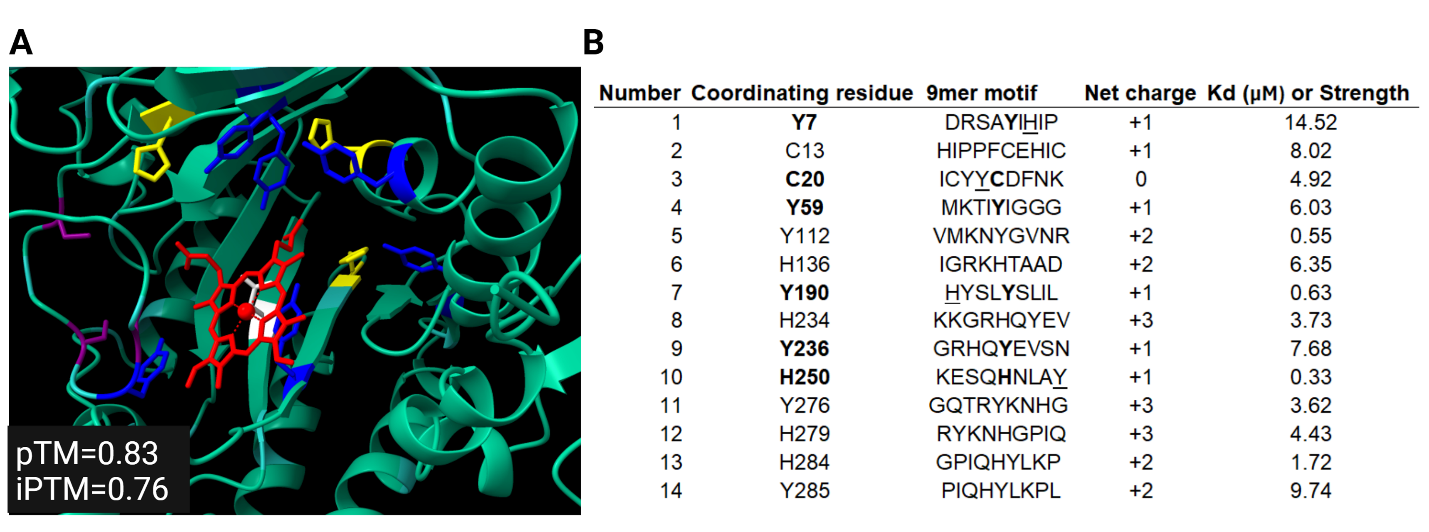


**Fig S1.** Analysis of RS05575 capacity to bind heme. (**A**) AlphaFold server was used to predict the structure when bound to heme. The cysteines of the CXXXCXXC (rSAM) motif are shown in purple, the conserved Asp159 involved in ChuW activity is shown in white, and histidine and tyrosine residues potentially involved in heme coordination are shown in yellow and blue respectively. Predicted Template Modeling score (pTM) specifies the accuracy of the structure where a score above 0.5 indicates a likelihood of the prediction being similar to the true structure. The interface Predicted Template Modeling score (ipTM) depicts the accuracy of the relative positions of the subunits of the predicted structure. A score >0.8 indicates high-quality predictions, between 0.6-0.8 indicates medium-quality predictions, and <0.6 indicates a low-quality prediction. Structure was modeled using ChimeraX 1.3. Blue= histidine, Yellow= tyrosine, and Purple=cysteine. (**B**) The HeMoQuest webserver was also used to identify putative heme binding residues. The coordinating residue indicates the amino acid likely to directly interact with heme, the nonapeptide motif is the sequence surrounding the heme coordinating residue, and the K_d_ values are the predicted binding affinity for the nonapeptides. <1 µM = good binding prediction, 1-5 µM = medium binding prediction, 5-10 µM = weak binding prediction, >10 µM = no biding prediction. Bolded coordinating residues and underlined residues in the nonapeptide motif designate residues that also appear to interact with heme in the predicted structure.


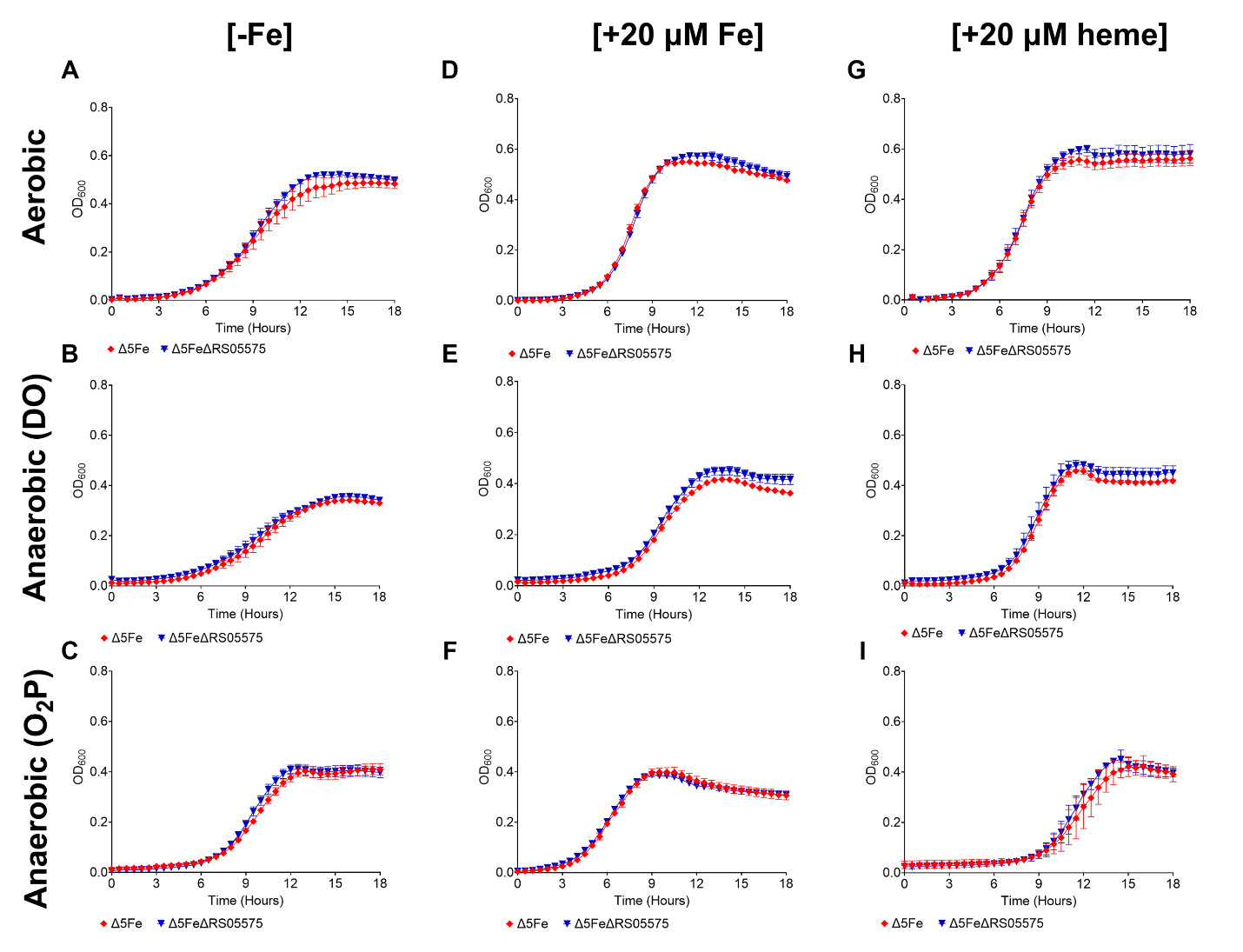


**Fig S2.** Growth of Δ5Fe and Δ5FeΔRS05575 in (**A**) FMC[-Fe], (**B**) FMC[+20 µM Fe], (**C**) FMC[+20 µM heme], (**D**) FMC_DO_[-Fe], (**E**) FMC_DO_[+20 µM Fe], (**F**) FMC_DO_[+20 µM heme], (**G**) FMC_O2P_[-Fe], H) FMC_O2P_[+20 µM Fe], and (**I**) FMC_O2P_[+20 µM heme]. Growth was monitored by measuring OD_600_ every 30 minutes using an automated growth reader. Error bars denote standard error of the mean from two independent experiments with three biological replicates.
